# Antioxidant activity of polyphenolic myricetin in vitro cell- free and cell-based systems

**Published:** 2016-06

**Authors:** Abolfazl Barzegar

**Affiliations:** Research Institute for Fundamental Sciences (RIFS), University of Tabriz, Tabriz, The School of Advanced Biomedical Sciences (SABS), Tabriz University of Medical Sciences, Tabriz, Iran

**Keywords:** Myricetin, ROS, Antioxidant, MTT, FRAP

## Abstract

Myricetin (Myc) is one of the most important flavonoids in diet due to its abundance in foods with the highest antioxidant activity. The antioxidant activity of Myc was studied in cell-free and cell-based systems to evaluate the ROS protection efficiency of Myc. The studies were based on the assessment of reducing power of Myc according to ferric ion reduction and intracellular ROS level measurement by assaying the cellular fluorescence intensity using dichlorodihydrofluorescein (DCF) probe as an indicator for ROS in cells. Moreover, the antitoxic capability of Myc was assessed using MTT method. Data indicated that intracellular ROS are highly toxic and applying low concentration of Myc not only inhibited cellular ROS production but also was accompanying with the protection of cells against the highly toxic and the lethal effects of peroxide compounds. Because of strong correlation between cellular ROS and their cell toxic properties, the higher antioxidant potency of Myc in cell medium resulted in effectively blocking intracellular ROS and protecting cell death. This property is achieved by the help of high polar solubility and cell membrane permeability of Myc.

## INTRODUCTION

Flavonoids compounds are a diverse group of plant metabolites and are found in a wide variety of human foods [1, 2]. The flavonoids, as a large and complex group, are the best defined groups of polyphenols in the human diet. They are a various and complex group of compounds that are absorbed in the gut and linked to human health [1]. They contain a three-ring structure with two aromatic centers and a central oxygenated heterocycle [3, 4]. Flavonoids can suppress carcinogenesis in animal models and there is considerable interest in the biological effects of these compounds at the cellular level [5]. Flavonoids are categorized into subgroups based on their chemical structure: flavanones, flavones, flavonols, flavan-3-ols, anthocyanins and isoflavones.

**Figure 1:**
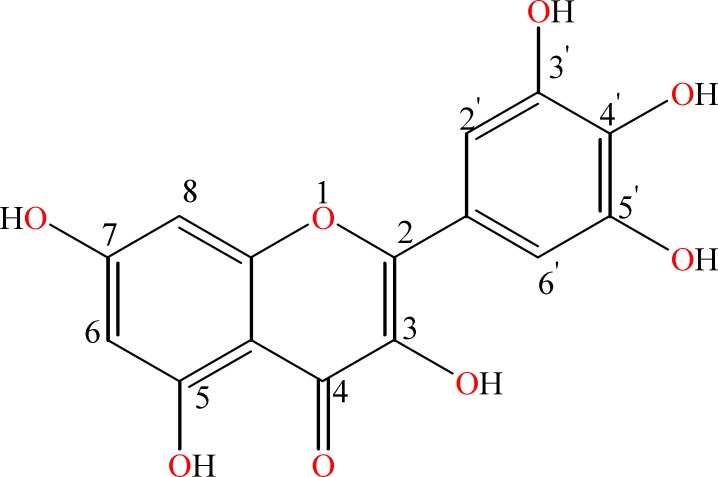
The chemical structure of myricetin (Myc)

Over 10,000 polyphenol compounds have been identified until now. However, only few of them have been investigated in detail [5]. Most common flavonoids include resveratrol, epigallocatechin 3-gallate, tyrosol, hydroxytyrosol, kaempferol, quercetin and myricetin (Myc) [6-8]. Myc is a natural flavonol that exists in tea, different vegetables, onions, berries, grapes and medical plants, with a unique chemical structure [7]. Myc consists of two aromatic rings linked together with a heterocyclic pyrone ring as displayed in Figure 1. It induces pancreatic cancer cell death [9], inhibits DNA strand breakage [10], attenuates the ultraviolet B induced COX-2 expression and skin tumor formation in a mouse skin model [11]. Treatment of insulin-resistant rats with Myc leads to the alteration in the phosphorylation of the insulin receptor, with subsequent effects on glucose-transporter subtype 4 translocation [12]. Results of *in vitro *studies suggested that high concentrations of Myc can cause change in LDL cholesterol level via an increase in the uptake of LDL cholesterol by white blood cells. A study correlated high Myc consumption with lowered rates of prostate and pancreatic cancers [13, 14]. Consequently, the compound Myc has a significant pharmacological importance as a medicinal agent. The action of Myc at the molecular level is mainly based on antioxidant effects. The antioxidant and radical scavenging activities of Myc have been widely studied by different researchers for many years [15-18]. Results of previous studies have showed that each Myc molecule is capable of scavenging different radicals and it was more effective than *α*-tocopherol as an antioxidant in liposomes [15]. However, the intracellular antioxidant behavior of Myc has not studied yet.

In this study, the ability of Myc to reduce and scavenge intra-cellular ROS was evaluated using human MCF-7 cells as an *in vitro *cell model. In addition, cell-free system including “reducing power” has been applied for evaluating the effect of polar solvent in ferric ion reduction capability of Myc.

## MATERIALS AND METHODS

**Materials: **Chemical compounds including Myc, trichloroacetic acid, and cumene hydroperoxide (CHP) were purchased from Sigma Chemical Co. Potassium ferricyanide and ferric chloride were acquired from Merck. 2',7'*-*dichlorodihydrofluorescein diacetate (DCFH2-DA) was obtained from Molecular Probes (Eugene, OR). Fetal bovine serum was from GIBCO (Grand Island, NY). RPMI 1640, antibiotics and sterile plastic for cell culture were from Flow Laboratory (Irvine, UK). Water treated in a MicroMed-TKA system (conductivity < 0.1 µS cm-1), was used to prepare the solutions. Stock solution of Myc was prepared using dimethylsulfoxide (DMSO) as a solvent from Merck. Human MCF-7 breast cancer cells were obtained from the pharmaceutical nanotechnology research center, Tabriz University of Medical Sciences, Tabriz, Iran

**Cell cultures: **Human MCF-7 breast cancer cells were grown in RPMI 1640 supplemented with 10% (v/v) fetal bovine serum, 100 U mL−1 penicillin, and 100 mg mL−1 streptomycin. These cells were incubated at 37 ˚C with 5% CO2 and usually sub- cultured once every 3 days.

**DCF method for detection of the intracellular ROS: **The intracellular ROS level in MCF-7 cells was measured using DCF method. The details of DCF method were clearly explained in our previous publications [19-21]. Briefly, the cell samples were incubated one hour in the presence of 5 µM DCFH2-DA, followed by two times washing with PBS. The washing procedure has been applied by the help of centrifugation with 2000 rpm to remove the extracellular DCFH2-DA. The oxidation of DCFH2-DA by intracellular ROS resulted in fluorescent DCF which stains the cells. Hence, the intracellular ROS generation of cells can be investigated using DCF method as an indicator to detect and quantify intracellular produced reactive oxygen species. The trapped fluorescent DCF dye inside the cells was used to evaluate and detect intracellular ROS by spectrofluorometer. The intracellular ROS generator compound, CHP, was used in 300 µM concentration. The incubation time for CHP was 2 min followed by the fluorescent intensity changes by adding different concentrations of Myc (0.0, 0.05, 0.1 and 0.2 µM). Experiments were done in triplicate and the mean value was recorded.

**MTT assay: **In order to assay the antitoxic capability of Myc, methylthiazole tetrazolium (MTT) method was utilized [19]. Viable cell numbers were recorded by measuring the absorbance at a certain wavelength of 500–600 nm. MCF-7 cells were seeded into 6-well plates until they reached 95–100% confluency. Then, they were incubated for 30 min with different concentrations of Myc. Afterward, cells were induced with cumene hydroperoxide (CHP) as a powerful cytotoxic ROS-generating compound [21, 22]. The cells were incubated with MTT at a final concentration of 1mg/mL at 37 ˚C for one hour. Cells were separated by trypsinization followed by centrifugation at 1200 rpm for 5 min. Then, the pellet was re-suspended in 300 mL phosphate-buffered saline (PBS) and sonicated on ice for 20 seconds with an Ultrasonic W-225R, at setting 4, and centrifuged in a microfuge at 13000 rpm for 10 min. In the final step, the supernatant was discarded and the water-insoluble formazan assay product was dissolved in DMSO and measured at 560 nm.

**Ferric ion reducing antioxidant power (FRAP assay): **FRAP activity was measured according to our previously published method [21, 22]. The process by which the ferric ion reduces into the +2 oxidation ferrous ion state (Fe2+) is known as reducing power assessment. The reducing power of Myc was determined by analyzing its electron donor potency according to ferric ion reduction. Potassium ferricyanide (1%) was shortly incubated with different concentrations of Myc for 30 min at 50 °C in phosphate buffered saline (PBS), then FeCl3 (0.1%) and trichloroacetic acid (10%) were added and mixed. After the addition of trichloroacetic acid (10%) and FeCl3 (0.1%) the absorbance at 700 nm was recorded as reducing power of Myc in PBS solution. Samples with greater reducing power showed higher absorbance at 700 nm.

## RESULTS

**Figure 2:**
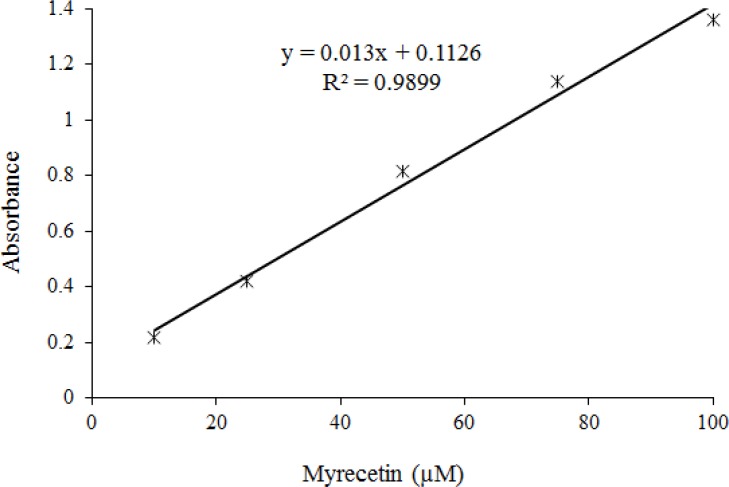
Ferric iron (Fe3+) reduction capacity of Myc. The absorbance at 700 nm was recorded as a function of Myc concentrations in phosphate buffered saline (PBS) to monitor the reduction of ferric ion to ferrous ion

Electron-transfer reaction of Myc has been focused on Fe+3 reduction to Fe+2 in aqueous solution that can be a significant indicator of the antioxidant activity known as ferric ion reducing antioxidant power (FRAP). The FRAP assessment provides clear information about the electron transfer potency of an antioxidant which is a simple, rapid, and relatively inexpensive assay [22]. The absorbance at 700 nm was recorded as a function of Myc concentrations in phosphate buffered saline (PBS) to monitor the reduction of ferric ion to ferrous ion. Figure 2 indicates a significant linear relationship between Myc concentrations and reduced amounts of iron (III) ions in polar solution.

**Figure 3:**
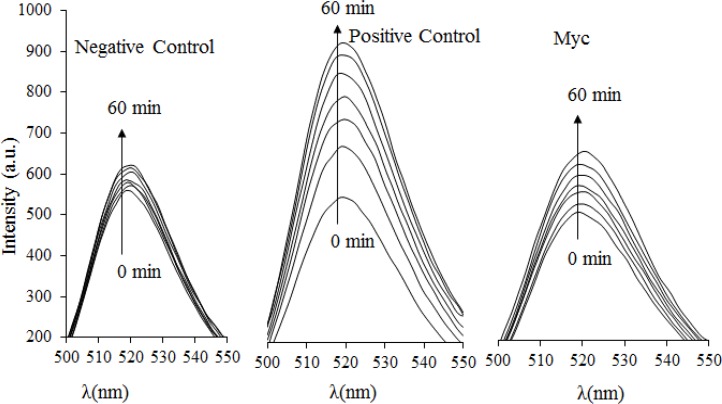
Intracellular ROS determination by DCF method. The changes of fluorescence spectra were monitored for the intracellular ROS during 60 min. The excitation wavelength, excitation slit and emission slit were 485, 5 and 10 nm light path respectively. Samples were negative control (without having antioxidant and ROS stimulator), positive control (having intracellular ROS inducer agent of cumene hydroperoxide,CHP) and myricetin (having intracellular ROS inducer agent of CHP plus 0.5µM myricetin).

The ability of Myc to scavenge intracellular ROS was investigated using MCF-7 cells based on the DCF method. CHP is one of the oxidizing agents which has been used as an intracellular source of reactive oxygen intermediates [19]. Myc combats intracellular ROS generation which leads to decrease in the amount of DCF fluorescence, as shown in Figure 3. In the absence of Myc, CHP causes substantial oxidation of DCFH2 to DCF, leading to an increased rate of fluorescence intensity change. Addition of Myc suppresses the intracellular fluorescence intensity at 485 nm during 60 minutes. Consequently, the efficient suppression of intracellular ROS production by Myc indicates that this compound enters the cells and acts with strong radical scavenging potency in the polar intracellular environment. Moreover, the cell membrane permeability of Myc was evaluated based on simultaneous DCF fluorescence changes that are indicated in Table 1. More importantly, we observed an immediate decrease in DCF fluorescence after the addition of 0.1 and 0.2 μM Myc which implies the rapid penetration of Myc into cells and suppression of ROS generation. As a result, Myc is able to diffuse through the cell membrane into the cells, where it prevents the production of different ROS compounds.

**Table 1:** The membrane permeable efficiency of Myc to reduce intracellular ROS

**Samples(µM)**	**ΔF1**	**ΔF2**	**ΔF3**	**ΔF4**
**0**	81	88	150	121
**0.05**	70	80	120	100
**0.1**	46	54	80	68
**0.2**	38	45	70	60

**Note: **Cell samples were induced by 300 µM radical generator CHP at the same time following the addition of

different concentrations of Myc (0.0, 0.05, 0.1 and 0.2 µM). DCF fluorescence changes were evaluated for 10 minutes and reported as ΔF/10 min for four different independent assays (ΔF1, ΔF2, ΔF3, ΔF4). High ΔF values denote high intracellular ROS. None of the Myc samples tested, rose to fluorescence on their own.

**Figure 4:**
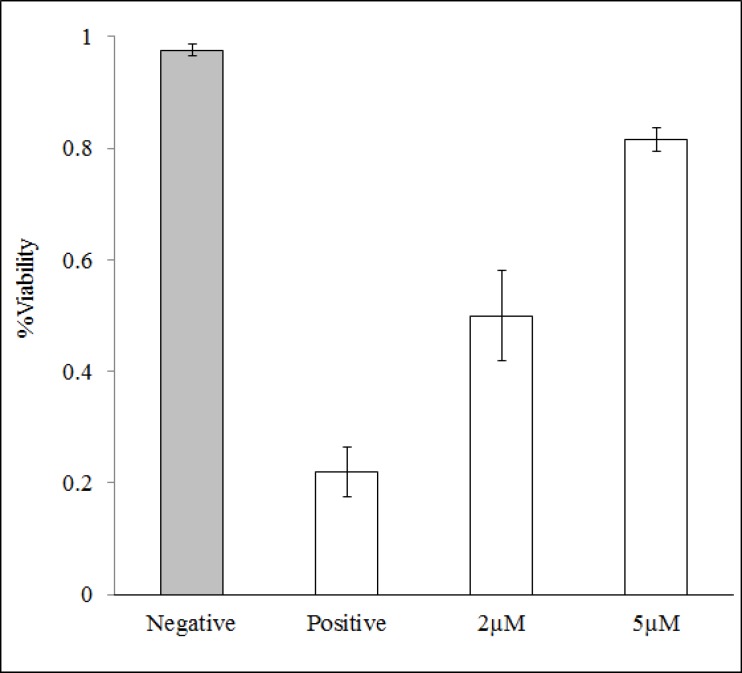
Cell viability assay using MTT method. Cells were treated with 5 µL (1:100) CHP. Negative control indicates no addition of CHP/antioxidant and positive control denoting only presence of CHP. Treating cells by 2 µM and 5 µM Myc during 30 min led to the decrease followed by complete suppression of the toxic effects of CHP.

MTT method was used to evaluate the antitoxic properties of Myc against cytotoxic and the lethal effects of CHP. As saying the antitoxic property of Myc has been performed based on the suppression of cytotoxic effects of CHP. The reduction of yellow MTT to purple formazan takes place only when mitochondrial reductase enzymes are active, and therefore the amount of conversion can be directly accounted for the percentage of viable (living) cells [19]. Figure 4 shows that in the absence of CHP, 95% of cells are viable that reduce MTT compound. While, CHP is a highly toxic and lethal compound for MCF-7 cells that causes 75% of cells to die. The presence of Myc in samples almost suppressed the lethal effects of CHP. Hence, the role of Myc in blocking the toxic and lethal effects of CHP has a strong correlation with its intracellular ROS scavenging potency, as it was mentioned in Figure 3.

## Discussion

Reactive oxygen species, including free radicals, are formed by exogenous chemicals and endogenous metabolic processes in the human body. Different diseases have been associated with excessive ROS (Fig. 5), which are produced mostly in the mitochondria as byproducts of cell respiration during mitochondrial electron transport and other metabolic reactions [23, 24]. The suppressing/inhibiting intra-cellular ROS generation is the main challenge for preventing and probably cure the oxidative stress or diseases mentioned in Figure 5. Our data clearly show that Myc has excellent behavior to scavenge free radicals inside the cells. *In vitro *cell-based and cell-free systems studies confirmed this antioxidant is active both in extracellular and intracellular media that reduces ferric ions and cellular ROS, respectively. FRAP assay showed a higher reducing capability of Myc because of its unique structure and functional groups, including pyrone and two phenyl rings and with more π electrons (see Fig. 1). The π electrons can fully conjugate between rings; this endows Myc with a high reducing capability. Therefore Myc is an antioxidant that is active in polar PBS solvent. Two important findings from intracellular ROS assay showed that; a) Myc easily penetrates from cell membrane into cytoplasm and effectively scavenges the produced ROS in the polar environment. The results of this experiment were confirmed by FRAP assay. b) The role of Myc in blocking the toxic and lethal effects of CHP has a strong correlation with its intracellular ROS scavenging potency. The ROS and MTT results are consistent and show highly radical chain breaking potency of Myc. These data confirmed that Myc could be an active antioxidant to protect tissues against free oxygen radicals in polar environment of body such as cerebrospinal fluid, interstitial fluid, plasma, and inside/outside of cells. Consequently since ROS contribute to a broad range of pathologies and many of the implicated diseases such as cancers, cardiovascular diseases, and neurological diseases, leading causes of death, consuming high levels of flavonoid Myc antioxidant-rich fruits could help ones to combat with the ROS- associated diseases resulted in better health.

Most antioxidants are known radical chain breaking reactions based on their potency in cell-free systems. Antioxidants preferentially localize to cellular compartments based on solubility. Unfortunately, most of them are not effective within the cells, mainly because of limited solubility, low permeability, and self-toxic lethal effects. The different properties of Myc such as cell protection efficiency against toxic CHP, intracellular ROS inactivator and ferric ion reduction power in polar medium proposed the efficient flavonoid for the potential clinical, biological, and biotechnological applications.
